# Schmallenberg virus in Germany 2011–2014: searching for the vectors

**DOI:** 10.1007/s00436-015-4768-5

**Published:** 2015-10-13

**Authors:** Daniela Kameke, Doreen Werner, Bernd Hoffmann, Walburga Lutz, Helge Kampen

**Affiliations:** Institute of Land Use Systems, Leibniz Centre for Agricultural Landscape Research (ZALF), Eberswalder Str. 84, 15374 Müncheberg, Germany; Institute of Infectology, Friedrich Loeffler Institute (FLI), Südufer 10, 17493 Greifswald, Germany; Institute of Diagnostic Virology, Friedrich Loeffler Institute (FLI), Südufer 10, 17493 Greifswald, Germany; Landesamt für Natur, Umwelt und Verbraucherschutz, Forschungsstelle für Jagdkunde und Wildschadenverhütung (FJW), Pützchens Chaussee 228, 53229 Bonn, Germany

**Keywords:** SBV, Ceratopogonidae, *Culicoides*, Biting midges, Obsoletus complex, Simuliidae

## Abstract

Following the emergence of Schmallenberg virus (SBV) in 2011, 21,397 culicoid biting midges (Diptera: Ceratopogonidae) from targeted and non-targeted sampling activities carried out during the summer months of 2011 to 2013 and in late 2014 in various regions in Germany were analyzed for the virus by real-time RT-PCR. While no SBV was found in biting midges collected during 2011 and 2013, 2 out of 334 pools including 20 and 22 non-engorged females of the Obsoletus complex sampled in 2012 tested positive for the SBV S-segment with C_t_ values of 42.46 and 35.45. In addition, 673 black flies (Diptera: Simuliidae) captured during the same studies were screened for the presence of SBV and proved negative. In late autumn 2014, biting midges were collected again in a limited study in eastern Germany after some cases of SBV infection had occurred in a quarantine station for cattle. Due to the unfavorable seasonal weather conditions, only few specimens were caught, and these were also negative for SBV. The German experience suggests that biting midge collections launched only after an outbreak and are not locally targeted may be ineffective as to virus detection. It rather might be advisable to collect biting midges at sentinel farms on a permanent basis so to have material available to be examined in the case of a disease outbreak.

## Introduction

In late summer 2011, Schmallenberg virus (SBV), a new Orthobunyavirus (family Bunyaviridae) emerged in Germany. SBV infects ruminants and causes a mild clinic with possible symptoms such as diarrhea, fever, or a decrease in milk production. Infection of pregnant animals can lead to miscarriages, stillbirths, or severe deformations of the unborn (Hoffmann et al. 2012). Once infected, hosts are believed to develop immunity (Conraths et al. 2013a). A zoonotic potential seems unlikely but could not be completely ruled out (Ducomble et al. 2012; Reusken et al. 2012). Based on the close relationship of SBV to well-known viruses such as the Akabane virus (family Bunyaviridae, genus Orthobunyavirus), which is widely distributed in Africa and Asia and transmitted by Ceratopogonidae and Culicidae, it was hypothesized that these groups of arthropods could also function as possible vectors of SBV (Hoffmann et al. 2012; Garigliany et al. 2012). While studies could not confirm the involvement of mosquitoes (Scholte et al. 2014; Wernike et al. 2014; Manley et al. 2015), SBV was soon found within culicoid biting midges of the Obsoletus complex (Rasmussen et al. 2012). Further studies confirmed these findings and detected the virus in *Culicoides obsoletus* (Meigen), 1818; *C. chiopterus* (Meigen), 1830; *C. dewulfi* Goetghebuer, 1936; *C. scoticus* Downes & Kettle, 1952; *C. punctatus* (Meigen), 1804; *C. pulicaris* (Linnaeus), 1758; *C. nubeculosus* (Meigen), 1830; and *C. imicola* Kieffer, 1913 (e.g. De Regge et al. 2012, 2014; Elbers et al. 2013; Larska et al. 2013a, 2013b; Balenghien et al. 2014).

Additionally, specimens belonging to laboratory colonies of *C. nubeculosus* and the North American species *C. sonorensis* Wirth & Jones, 1957 were found to reproduce the virus upon feeding on a viraemic blood source and facilitate dissemination into the salivary glands under experimental conditions (Veronesi et al. 2013).

While most SBV-infected midges were collected between early August and late October (De Regge et al. 2012, 2014; Rasmussen et al. 2012, 2014; Elbers et al. 2013; Larska et al. 2013a, 2013b), Elbers et al. (2015) detected the virus in 2012 in culicoids sampled already in July. Probably due to the Mediterranean climate, SBV was furthermore present in catches made in Italy as early as May suggesting the likelihood of SBV overwintering in midges (Goffredo et al. 2013) and as late as November (Goffredo et al. 2013; Balenghien et al. 2014).

The involvement of black flies (Simuliidae) in the transmission of SBV has never been investigated before. Simuliids are closely related to the Ceratopogonidae and include known vectors of nematodes. By contrast, only few viruses have been detected in Simuliidae so far (Braverman 1994; Smith et al. 2009), which might be attributed to the scarce number of studies conducted. Therefore, an involvement of simuliids in the transmission of SBV cannot be ruled out. Also, the rapid geographic spread of SBV in 2011 and 2012 indicated that arthropods other than Ceratopogonidae might have been involved in the transmission of the pathogen (Goffredo et al. 2013).

Several European countries were affected by SBV during its first transmission season in 2011 (Conraths et al. 2013a). During the vector season in 2012, the disease re-emerged in countries already affected and continued to spread to other yet uninvolved European countries (Conraths et al. 2013b). In 2013, the number of new SBV infections decreased significantly in countries previously affected, a tendency that continued in 2014.

**Fig. 1:**
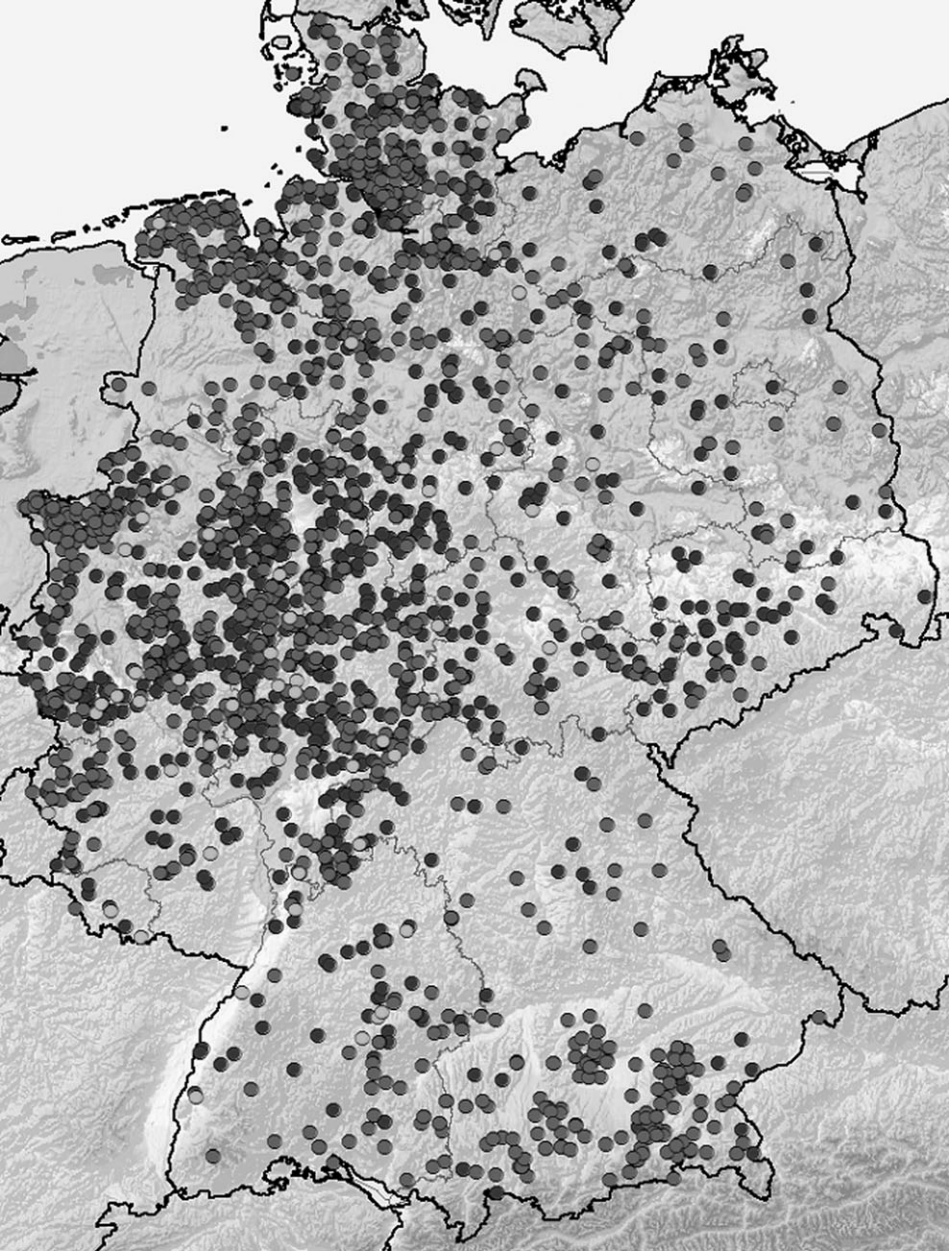
Reported cases of SBV infection in Germany as of 22 January 2013. *Dark-gray dots*: cattle, *black dots*: sheep, *light gray dots*: goats (source: FLI Archive. Maps of the distribution of ‘Schmallenberg virus’ in Germany, www.fli.bund.de/no_cache/de/startseite/aktuelles/tierseuchengeschehen/schmallenberg-virus/archiv-der-karten-2013.html)

In Germany, SBV activity had its peak in 2012. Southern and eastern regions of the country were much less affected at that time than western, central, and northern regions (Fig. 1). During 2013, the number of new infections decreased significantly, and the main viral activity took place in southern Germany (federal state of Bavaria). In 2014, only a handful of new cases were registered at the beginning of the year, and SBV seemed to have disappeared. However, in October 2014, new cases emerged in several German localities (Wernike et al. 2015). Some of them were related to an open quarantine station near the city of Cottbus, eastern Germany, where several cattle were proven to be freshly infected by SBV.

The objectives of this paper were I) to analyze the level of SBV circulation in culicoid biting midges in Germany for 2011, 2012 and 2013; II) to identify potential SBV vector species in Germany; and III) to check for the presence of SBV-infected culicoid biting midges near a single stable with acute viral activity in late 2014.

## Materials and methods

### Collection and identification of culicoid biting midges

During the summer months of the years 2011, 2012, and 2013, regular insect collections were done using BG sentinel UV-light suction traps (BG-S) and Onderstepoort Veterinary Institute UV-light suction traps (OVI traps).

**Fig. 2:**
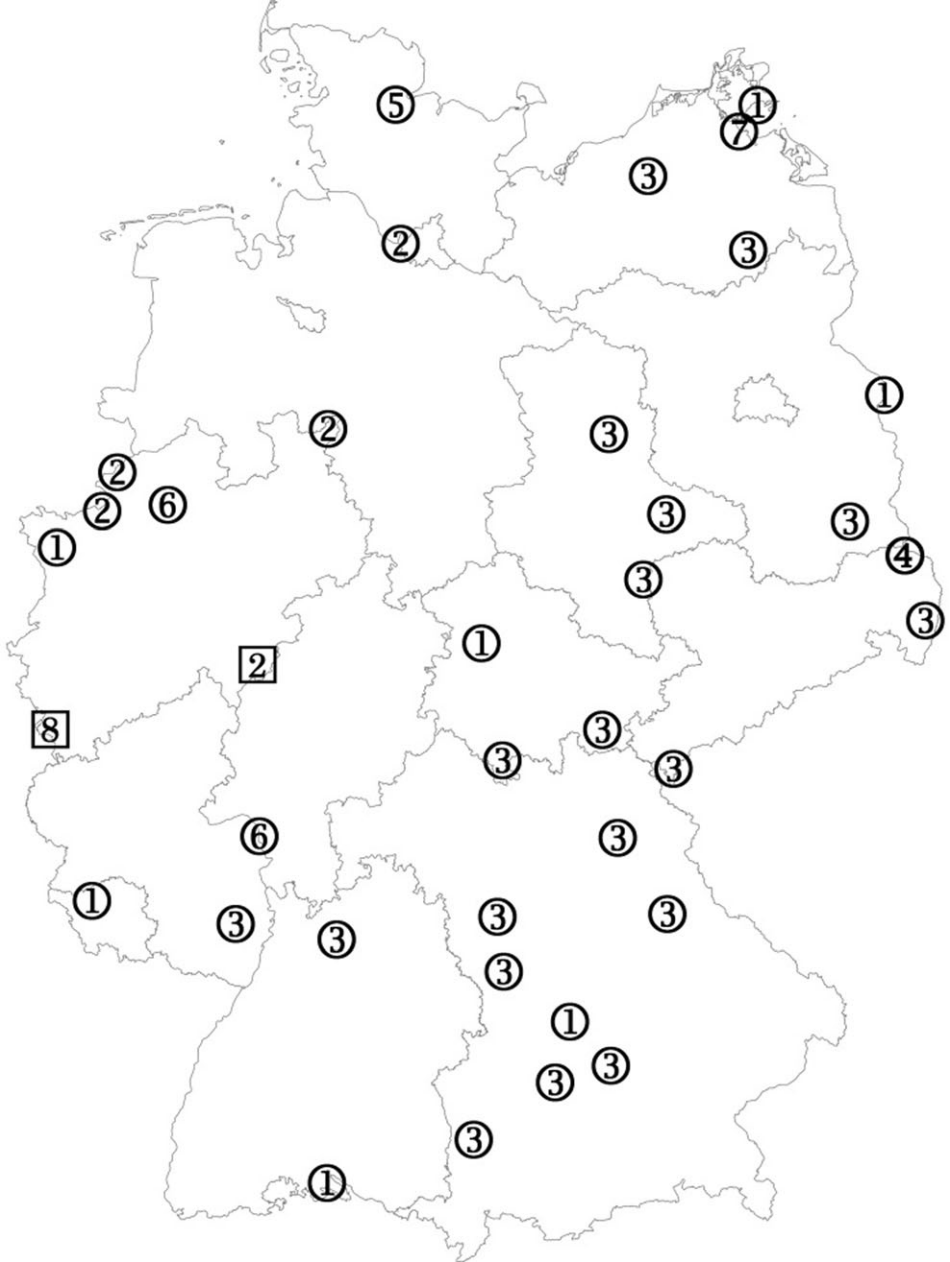
Collection sites and SBV findings during the various collection years. *1*, 2011; *2*, 2012; *3*, 2013; *4*, 2014; *5*, 2011–2013; *6*, 2011 + 2012; *7*, 2011 + 2013; *8*, 2012 + 2013; negative for SBV (*enclosed in circle*), positive for SBV (*enclosed in square*)

In 2011 and 2012, the collections were not specifically targeted at ceratopogonids or simuliids as potential vectors of disease agents and only some of the traps were operated inside, or in close proximity to, animal shelters harboring potential infective blood hosts such as cattle, sheep, or goats. By contrast, most of the traps operated in 2013 were set up on farms keeping ruminants. Two insect traps were installed for several weeks close to a quarantine stable for cattle soon after the occurrence of acute SBV infections in 2014. All traps (Fig. 2) were run once a week for approximately 24 h unless stated otherwise.

2011 and 2012, BG-S traps were operated between April 21 and October 22, 2011 and between May 20 and November 3, 2012 at 10 collection sites per year. As three sites remained identical over the two years, 17 sites were sampled altogether. The distribution of the collection sites throughout Germany was as follows: nine in western Germany, one in central Germany, two in southern Germany, one in eastern Germany, two in north-eastern Germany, and two in the northern parts of the country (Fig. 2).

2013, BG-S and OVI traps were operated between April 19 and October 25 at 22 collection sites throughout Germany. Nineteen sampling sites were located in the southern, eastern, and north-eastern parts of Germany, two sampling sites in western and one sampling site in northern Germany (Fig. 2).Three of the locations had already been utilized in at least one of the previous years. During the last week of January, three additional BG-S were set up inside a sheep stable with fresh cases of acute SBV infections (Wernike et al. 2013).

2014, Two OVI traps were operated between November 7 and December 18 at two collection sites near a quarantine stable with acute cases of SBV infection in cattle (approximately 40 km southeast of Cottbus, close to the Polish border) (Fig. 2). Both OVI traps were run on a daily basis.

All insects were collected in 75 % ethanol until further investigation under a stereo microscope (Olympus SX12). Based on their wing maculation, female culicoids were morphologically identified to complex or species level following the keys of Delécolle (1985) and Mathieu et al. (2012). In this paper, references to the “Obsoletus complex” include the species *C. obsoletus* s.s., *C. scoticus*, *C. chiopterus*, and the isomorphic species *C. dewulfi*.

### Insect pools

For virus detection, female *Culicoides* Latreille, 1809 were pooled according to species or species complex as well as collection site and date, with up to 50 specimens per pool. Blood-fed individuals were pooled separately following the same criteria.

Additionally, Simuliidae were pooled according to sampling site and date but without further identification. Pool sizes ranged from 1 to 25 specimens. Again, blood-fed individuals were pooled separately following the same criteria.

### Detection of SBV genome

Viral RNA was extracted using the NucleoSpin 96 Virus Core Kit (Macherey-Nagel) following the manufacturer’s instructions. Eluates were then tested by the SBV-S3 one-step real-time RT-PCR as described by Bilk et al. (2012) and Hoffmann et al. (2012). As part of a duplex RT-qPCR, the assay was combined with internal control assays: a universal internal control system IC2-RNA (Hoffmann et al. 2006) and the co-amplification of a housekeeping gene, beta-actin (Toussaint et al. 2007).

Weak positive results with C_t_ values over 35 were retested to confirm positivity of the samples. Retesting was performed with the S3- and the L1.4-RT-qPCR assay (Fischer et al. 2013). If both assays showed positive results, the pool was considered SBV-positive. If the less sensitive L-amplicon could not be detected, the retest was repeated following a new extraction. The sample was still considered positive if the S3-amplicon could be detected in every (re)test.

## Results

A total of 21,397 culicoid biting midges from 171 samples were divided into 945 species-specific pools (average pool size = 22.64 specimens), including 110 pools containing 2110 blood-fed individuals (Table 1). While 19,107 of the midges belonged to the Obsoletus complex, the most abundant species complex, 2261 individuals were members of the Pulicaris complex and 29 specimens belonged to other culicoid species.

**Table 1:** Species/species group and numbers of female *Culicoides* and Simuliidae specimens and species composition tested for SBV including number of examined pools (in parentheses) and blood-fed individuals (in bold)

Year	*Culicoides*	Determined as “Obsoletus complex”	Determined as “Pulicaris complex”	Specimens identified to species level	Identified species [numbers of specimens]	Simuliidae
2011	4999 (218) **32 (12)**	4042 (105) **22 (6)**	952 (110) **5 (3)**	5 (3) **5 (3)**	*C. achrayi* Kettle & Lawson, 1955 **[3]** *C. circumscriptus* Kieffer, 1918 **[1]** *C. riethi* Kieffer, 1914 **[1]**	633 (37) **0**
2012	5562 (334) **72 (22)**	5197 (265) **54 (9)**	327 (53) **5 (3)**	38 (16) **13 (10)**	*C. achrayi* Kettle & Lawson, 1955 **[4]** *C. clastrieri* Callot, Kremer & Deduit, 1962 **[2]** *C. grisescens* Edwards, 1939 [19] *C. impunctatus* Goetghebuer, 1920 [1] *C. newsteadi* Austen, 1921 [5] *C. picturatus* Kremer & Deduit, 1961 **[1]** *C. salinarius* Kieffer, 1914 **[1]** *C. segnis* Campbell & Pelham-Clinton, 1960 **[2]** *C. truncorum* Edwards, 1939 **[3]**	3 (2) **2 (1)**
2013	10,803 (385) **2006 (76)**	9836 (243) **1973 (62)**	858 (118) **21 (8)**	109 (24) **12 (6)**	*C. achrayi* Kettle & Lawson, 1955 **[9]** *C. grisescens* Edwards, 1939 [7] *C. impunctatus* Goetghebuer, 1920 [5] *C. newsteadi* Austen, 1921 [85, **1**] *C. picturatus* Kremer & Deduit, 1961 [1] *C. vexans* (Staeger), 1839 **[2]** *C. obsoletus* (Meigen), 1818 [1]^a^	37 (9) **4 (2)**
2014	33 (8) **0**	32 (7) **0**	0 **0**	1 (1) **0**	*C. punctatus* (Meigen), 1804	0 **0**
Total	21,397 (945) **2110 (110)**	19,107 (620) **2049 (77)**	2137 (281) **31 (14)**	153 (44) **30 (19)**		673 (48) **6 (3)**

^a^Described by Wernike et al. (2013)

Additionally, 673 Simuliidae from 30 samples, predominantly collected between May and September 2011 (Table 2), were sorted and pooled as described above, creating 48 pools. Six of the black flies were blood-fed (Table 1).

**Table 2:** Sampling periods of *Culicoides* and Simuliidae tested for the presence of SBV

Year	2011		2012		2013		2014	
Month	*Culicoides*	Simuliidae	*Culicoides*	Simuliidae	*Culicoides*	Simuliidae	*Culicoides*	Simuliidae
January	n.d.	n.d.	n.d.	n.d.	1	0	n.d.	n.d.
April	137	0	n.d.	n.d.	917	0	n.d.	n.d.
May	331	189	1166	0	2486	3	n.d.	n.d.
June	401	112	667	1	2334	3	n.d.	n.d.
July	489	50	914	2	3159	31	n.d.	n.d.
August	2648	133	1822	0	1356	0	n.d.	n.d.
September	988	120	487	0	274	0	n.d.	n.d.
October	5	29	492	0	276	0	n.d.	n.d.
November	n.d.	n.d.	14	0	n.d.	n.d.	33	0
Total	4999	633	5562	3	10,803	37	33	0

*n.d.* not done

Among the 945 culicoid pools screened for SBV, two pools (0.2 %) were weakly positive for the pathogen. Both positive samples were collected in 2012 in western Germany, resulting in an average of 0.6 % (2/334) of positive pools for 2012. No *Culicoides* sampled during 2011, 2013 and 2014, and none of the Simuliidae tested positive for SBV.

Positive pool 1: The pool contained 20 specimens of the Obsoletus complex which had been sampled on September 3, 2012 in the German federal state of North Rhine-Westphalia (N 50° 57′ 40″, E 8° 19′ 16″) inside a barn housing a few sheep and goats. The pool showed a C_t_ value of 42.46 for the S3 region (positive control C_t_ = 27.23).

Retest C_t_ values were 37.36 and 38.23 (mean value = 37.795) for the S3-RT-qPCR and 36.96 and 37.41 (mean value = 37.185) for the L1.4-RT-qPCR.

Positive pool 2: The pool contained 22 specimens of the Obsoletus complex which had been sampled on August 29, 2012 in North Rhine-Westphalia (N 50° 31′ 55″, E 6° 19′ 21″) at a forester’s lodge. The pool showed a C_t_ value of 35.45 for the S3-RT-qPCR (positive control C_t_ = 27.07), and the retests delivered C_t_ values of 34.86 and 37.14 (mean value = 36). The L1.4-RT-qPCR reacted negative in all tests. The retest including re-extraction confirmed previous results with C_t_ values of 38.90 and 38.28 (mean value = 38.59) for the SBV-S3-RT-qPCR and negative results for the L1.4 assay.

## Discussion

In this study, more than 21,000 female culicoid biting midges collected between 2011 and 2014 were screened for SBV. Only 0.2 % of the tested pools were weakly positive for the pathogen which is a remarkably low percentage considering the high SBV activity in ruminants in 2011 and 2012 and compared to studies in adjacent countries like Denmark (Rasmussen et al. 2012, 2014), the Netherlands (Elbers et al. 2013, 2015), Belgium (De Regge et al. 2012, 2014), and Poland (Larska et al. 2013a, 2013b). A more detailed analysis of each year of collection revealed that in our study, no SBV-positive biting midge was caught in 2011 when SBV was discovered. Since all hosts must have been susceptible to the virus at that time, a relatively high level of virus circulation should have been assumed soon after the outbreak. Especially, due to the majority of the tested ceratopogonids of our study (*n* = 4130) being collected during the months of July–October 2011 (Table 2), the presumed peak season of transmission, a high chance of detecting positive midges was expected for 2011. This is especially true, as most of the insect traps were located in western Germany where the first cases of Schmallenberg disease had been observed. However, the assumed high level of virus circulation is not reflected in the number of *Culicoides* positive for SBV in the present study. As the sensitivity of RNA extraction and amplification with the used protocol was demonstrated previously (Hoffmann et al. 2009), a methodological reason is not considered plausible. A degradation of viral RNA by the time of processing to explain the lack of SBV-positive midges for 2011 in Germany is also rather unlikely since the insects were stored in 75 % ethanol and were in good optical condition when tested. It seems more reasonable to assume that the choice of collection sites is the main factor for the lack of SBV-infected midges captured. As all insect traps operated during 2011 and 2012 were part of a monitoring program with a different focus, many of them were not installed right on farms with blood hosts susceptible to SBV. This finding is in accordance with Rasmussen et al. (2014) where none of the midges, collected during 2011 at various untargeted sampling sites in Denmark, tested positive for SBV, whereas a previous Danish study showed infection rates of 9.1 % at only a few targeted collection sites sampled during the same year (Rasmussen et al. 2012). Since the flight radius of ceratopogonids is limited, with 2–3 km at most (Kluiters et al. 2015), the presence of potential blood hosts might be crucial in finding the pathogen and might therefore explain the lack of SBV-positive specimens.

In the Netherlands, Elbers et al. (2013) were able to find the virus in biting midges in 2011 with a relatively low percentage of infected pools (2.3 % = 14/610 pools, with 10 midges/pool). By contrast, other studies carried out in 2011 showed much higher percentages of positive *Culicoides* midges. While Rasmussen et al. (2012) found a mean value of 9.1 % (2/22 pools, with 5 midges/pool) of SBV-infected midges in Denmark, infection prevalences varied locally between 3.7 % (5/134 pools, mean pool size = 8.3 midges/pool) and 15.9 % (7/44 pools, mean pools size = 19.3 midges/pool), with a mean infection prevalence of 6.7 % (12/178 pools) in Belgium (De Regge et al. 2012).

Our study confirms the circulation of SBV in German *Culicoides* for 2012, when the disease had its peak in countries previously affected by the pathogen. The two positive pools in Germany in 2012 demonstrated SBV in females of the Obsoletus complex but failed to confirm other species/species groups to carry the virus or to add new species to the list of potential vectors. The C_t_ values of the SBV S-segments in both positive samples were rather high, 42.46 and 35.45, respectively. Both Larska et al. (2013b) and De Regge et al. (2014) presented bimodal distributions of C_t_ values which are believed to display SBV at a transmissible and sub-transmissible level. The high C_t_ values in our study suggest that both positive midges did not contain SBV at transmissible levels and that the virus did not replicate inside the insects.

However, other than the results of the present study and the high number of German SBV cases would indicate at that time, a decrease in SBV infection cases in *Culicoides* in adjacent Netherlands was shown for 2012 by Elbers et al. (2015). The percentage of biting midge pools containing SBV in the Netherlands dropped to1.5 % (2/130, with 50 midges/pool) in 2012 but was thus still much higher than the rate of virus detection in German *Culicoides* collected in the same year (0.6 % = 2/334 pools, with 3715 tested culicoids sampled between July and October, see Table 2). A similar decline became visible in Belgium, where the average SBV infection prevalence in midges had decreased from 6.7 % in 2011 to 3.6 % (35/973 pools, mean pool size = 19.3 midges/pool) in 2012 (De Regge et al. 2012, 2014). Seroprevalence data of potential blood hosts confirmed the decrease of SBV circulation during 2012 in Belgium, but also revealed that the pathogen was still circulating during summer and early autumn 2012 (Méroc et al. 2013).

In Denmark, the *Culicoides* sampled in 2012 displayed a mean SBV infection prevalence of 15.8 % (41/260 pools, mean pool size = 6.5 midges/pool) (Rasmussen et al. 2014) which was much higher than for other European countries in 2012 and for Denmark in the previous year (Rasmussen et al. 2012).

In 2013, all of our traps were operated on farms housing SBV-susceptible blood hosts of culicoids such as cattle, sheep and goats. The collection sites were mainly located in the southern and eastern parts of Germany (Fig. 2) where reported cases of SBV had been much lower before than in western Germany (Fig. 1). A shift of the region mostly affected by Schmallenberg disease to the east and south was therefore expected due to a relatively low herd immunity assumed among ruminants. The number of screened midges caught from July to October 2013 (*n* = 5065, see Table 2) was much higher than that of tested culicoids collected during the same time period of the previous year (*n* = 3715, Table 2). However, the total lack of SBV in ceratopogonids collected in 2013 suggests that the virus might have already spread across the southern and eastern parts of Germany and induced immunity in ruminants to a much higher degree than previously thought. Supporting this, interviews with livestock farmers indicated that newborns with clinical signs typical for SBV infection had frequently not been reported due to apprehended additional work and economic loss. Therefore, it seems quite possible that the actual number of SBV infections in southern and eastern Germany prior to 2013 was higher than reported, and that virus circulation in 2013 took place on a much lower level than expected.

To compare SBV infection rates in biting midges, the sampling periods of the various studies must be considered. In Denmark and France, culicoids were collected during a few days in October 2011 only (Rasmussen et al. 2012; Balenghien et al. 2014), while the sampling period lasted from August until early October 2011 in the Netherlands (Elbers et al. 2013). As a matter-of-fact, the collection periods of the studies set limits to a possible detection of SBV beforehand (Rasmussen et al. 2012; Balenghien et al. 2014; Elbers et al. 2013). Other studies sampled midges over considerably longer time periods, such as July to October 2011 (De Regge et al. 2012), May to September 2012 (Elbers et al. 2015), or May to November 2012 (De Regge et al. 2014), but only detected SBV-positive culicoids during the summer months July, August, and September (De Regge et al. 2012, 2014; Elbers et al. 2015).

Two studies investigating the occurrence of SBV in midges at various places of Poland included sampling periods from September to October 2011 and from April to October 2012. The first SBV-infected culicoids were detected only during the second year of the epidemic, namely from late August until late October 2012, following the introduction of SBV-positive bulls (Larska et al. 2013a, 2013b).

The two SBV-infected midges of our investigation were captured on August 29 and September 3, 2012, which is in accordance with SBV detection in other studies (e.g., Elbers et al. 2013; De Regge et al. 2014).

In 2014, only very few cases of SBV infections were recorded in Germany, and the disease appeared to slowly disappear from the scene. In autumn, however, several acute infections were diagnosed again in various parts of Germany, among them an open quarantine stable near Cottbus, federal state of Brandenburg, East Germany (Wernike et al. 2015). Even though the climate was relatively mild for that time of the year, the vector season was almost finished and the number of active biting midges was low, as demonstrated by the collections. None of the collected midges tested positive for SBV.

Despite the findings of several studies from northern Europe, in which SBV-positive midges could only be detected during the summer months, a transmission of SBV during the winter season cannot be excluded (Wernike et al. 2013). As there is no real biting midge-free period during the year (Mehlhorn et al. 2009), the risk of an infection might be limited during the cold season but is not absent.

Based on speculations on other arthropods possibly being involved in the transmission of SBV (Goffredo et al. 2013), it was checked whether simuliids carried the pathogen.

As none of the black flies, mainly sampled in the summer months of 2011 (Table 2), tested positive for the virus, and given the extremely low infection prevalence of biting midges, it is impossible to make a statement on a role this group of dipterans might have for the spread of SBV. Still, the involvement of hematophagous arthropods other than ceratopogonids such as biting flies or tabanids cannot be excluded at this moment.

Our findings suggest that the chances to detect SBV in the culicoid biting midge fauna are rather low outside highly epidemic periods of disease and in collections not done in close proximity to susceptible ruminants even within epidemic periods. Therefore, the establishment of a monitoring program seems useful in order to collect Ceratopogonidae at sentinel farms throughout the vector seasons and to have material available for screening in the case of a disease incident, be it Schmallenberg disease, bluetongue, or other ceratopogonid-borne diseases still to come.

The danger of a new SBV outbreak might presently be low. Over time, however, immune ruminants will be replaced by naive ones, and therefore, the declining herd immunity will increase the risk of a new epidemic (Méroc et al. 2013; Veldhuis et al. 2015).
